# Effects of dimming light-emitting diode street lights on light-opportunistic and light-averse bats in suburban habitats

**DOI:** 10.1098/rsos.180205

**Published:** 2018-06-06

**Authors:** Elizabeth G. Rowse, Stephen Harris, Gareth Jones

**Affiliations:** School of Biological Sciences, Life Sciences Building, University of Bristol, 24 Tyndall Avenue, Bristol BS8 1TQ, UK

**Keywords:** ALAN, bat activity, dimming, light-emitting diode street lights, light-opportunistic species, light-averse species

## Abstract

Emerging lighting technologies provide opportunities for reducing carbon footprints, and for biodiversity conservation. In addition to installing light-emitting diode street lights, many local authorities are also dimming street lights. This might benefit light-averse bat species by creating dark refuges for these bats to forage and commute in human-dominated habitats. We conducted a field experiment to determine how light intensity affects the activity of the light-opportunistic *Pipistrellus pipistrellus* and light-averse bats in the genus *Myotis.* We used four lighting levels controlled under a central management system at existing street lights in a suburban environment (0, 25, 50 and 100% of the original output). Higher light intensities (50 and 100% of original output) increased the activity of light-opportunistic species but reduced the activity of light-averse bats. Compared to the unlit treatment, the 25% lighting level did not significantly affect either *P. pipistrellus* or *Myotis* spp. Our results suggest that it is possible to achieve a light intensity that provides both economic and ecological benefits by providing sufficient light for human requirements while not deterring light-averse bats.

## 1. Introduction

Over the last 60 years, artificial light at night (ALAN) has increased globally on average by 6% per annum [1]. Although more prevalent in developed countries, ALAN is now considered a global threat because of increasing urbanization and industrialization in many developing countries [2,3]. ALAN is the result of a number of artificially lit sources, but street lights are one of the main contributors as they are installed in most towns and cities across the world [3,4].

Many local authorities across Britain are replacing old lighting stock such as low-pressure sodium (LPS) and high-pressure sodium (HPS) street lights with light-emitting diode (LED) street lights [5]. LED street lights offer a number of advantages over older lighting technologies, including increased energy efficiency, flexibility and longevity [6]. In Britain, LED lights are predicted to contribute up to 70% of the outdoor and residential lighting by 2020 [7]. As well as installing LED lights, many local authorities are implementing strategies to save money and reduce their carbon footprints, such as part-night lighting and dimming. It is relatively easy to employ dimming regimes with LED lights because they have a rapid on/off time [6,8]. Dimming levels can be implemented and adjusted remotely using a central management system (CMS) [3,9]. Dimming LED street lights is typically carried out by pulse-width modulation, which manipulates the duty cycle of a signal, so that the amount of ‘on’ time is reduced, but the spectral output of the light is unchanged [10,11].

Bats are a useful taxon to study the ecological impacts of light because they are nocturnal and their response to ALAN varies across species. A number of species are considered ‘light opportunistic’ as they feed on the large numbers of insects attracted to lights [12,13]: the attraction-by-insects hypothesis [14]. In Europe, these species are typically from the genera *Eptesicus*, *Nyctalus* and *Pipistrellus*. However, even light-opportunistic bats such as *Pipistrellus pipistrellus* will avoid lit areas when commuting in urban habitats, preferring to cross gaps in vegetation where there is little artificial light [15]. They also avoid lit areas when drinking at water sources [16].

Conversely, light-averse bats, such as those species from the genera *Myotis*, *Plecotus* and *Rhinolophus*, seem to be negatively affected by all types of street lighting. It is thought that because light-averse bats are often slower flying, more manoeuvrable species [17,18], they avoid light to reduce the risk of predation [19,20]. Many are also of conservation concern because their wing shape limits dispersal and movement [21], and hence they are particularly vulnerable to anthropogenic pressures such as urbanization and the associated ALAN. As dimming reduces both the light intensity of the street light and the amount of light distributed from the light source, it might create dark refuges that light-averse bats could use for commuting and foraging in urban areas [3].

There are many examples of artificial lighting affecting orientation, reproduction, communication and foraging in nocturnal taxa [22–26]. However, few studies have explored the biological impacts of varying light intensities. For example, the reproduction and survival of fruit flies, *Drosophila melanogaster,* are negatively affected by increased light intensity [27]. Increased light intensity also has a detrimental effect on the activity and melatonin level of great tits, *Parus major* [28] and activity patterns of blue tits, *Cyanistes caeruleus* [29], interrupts immune responses of Siberian hamsters, *Phodopus sungorus* [30], and Swiss Webster mice, *Mus musculus* [31], but does not affect sleep in *Parus major* [32].

Studies on the effects of light intensity on bat activity have highlighted that even low levels of ALAN have a detrimental effect on the activity of light-averse species [26,33]. Even when LED street lights were dimmed to a low level (mean 3.6 lux, range 2.90–4.86 lux), there were significantly fewer passes from the light-averse bats *Myotis* spp. and *Rhinolophus hipposideros* than on unlit nights [26]. However, dimming street lights to an intensity below 3.6 lux may not be feasible: street lights exist for human safety and if humans cannot see their surroundings clearly because the light intensity is too low, this nullifies the benefits of having street lights [26,34].

Our aim was to determine whether street light dimming regimes currently used by local authorities can have ecological benefits for bats as well as economic benefits. We tested the following two hypotheses:

(i) bat activity of the light-opportunistic bat *P. pipistrellus* will decrease at dimmed LED lights compared with undimmed LED lights owing to reduced insect abundance at dimmed street lights; and
(ii) bat activity of light-averse species from the genus *Myotis* will increase at dimmed LED lights compared with undimmed LED lights because the reduced light distribution will create dark refuges for light-averse bats to forage and commute.

## 2. Methods

### 2.1. Experimental design

Fieldwork took place between May and August 2015 at 21 sites using existing street lights in Hertfordshire, southeast England. Each site consisted of three lighting columns (lamp posts), which ran a series of lighting levels: 0%, 25%, 50% and 100% of the original output. These lighting levels refer to changes in duty cycle as described in the Introduction. Illuminance values for the four lighting levels are provided in the Results. As our aim was to assess the impacts of different street lighting levels, we used three adjacent lighting columns per site to ensure that a stretch of road (at least 60 m) was subjected to the same lighting level. The experiment ran for eight nights at each site, with the lighting level switching every two nights, i.e. each lighting level ran for two consecutive nights. The lighting schedules were randomized across sites to prevent any order effects, and sites were separated by at least 1 km to ensure the collection of independent samples. The lighting levels we used were representative of differing light intensities being employed by local authorities. Light levels were controlled using pulse width modulation by a sub-contractor of Hertfordshire County Council using a CMS.

**Figure 1.**
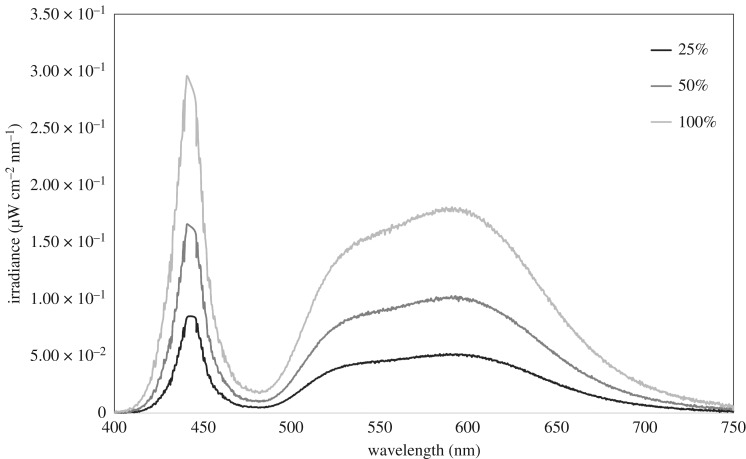
The spectral output of LED street lights at the three lighting levels (25%, 50% and 100%) from one of the 21 sites chosen at random.

All the street lights used in this study were neutral LED lights (MIDI, 97 W, 4250 K, Urbis Schreder, Basingstoke, RG24 8GG, UK) that were 10 m in height. We selected street lights along tree lines that contained trees more than 4 m in height, and each site was at least 20 m from the beginning of the tree line [15,35]. All sites were also close to other linear features such as hedgerows, and typical bat foraging habitats, such as woodland and grassland, were at least 35 m from a building, and were located on A (major) roads in suburban areas that experienced similar traffic intensity. To ensure that lighting levels were comparable across sites, both illuminance (lux) and irradiance (µW cm^−2^ nm^−1^) were measured. We used a TES 1330 lux meter (ATP Instrumentation Ltd, Ashby-de-la-Zouch, LE65 2UU, UK) at 1.8 m from the ground, directly underneath the lantern of the street light to measure illuminance, and a calibrated Ocean Optics USB 2000 spectrometer (Largo, FL 33777, USA), a 7 m P400-5-UV/VIS patch cord and a CC-3 cosine corrector, all positioned 5 m directly underneath the lantern, to measure irradiance. Irradiance readings also allowed us to ensure that the spectral output of the street light remained unchanged and that only intensity varied with each light level (figure 1).

We measured bat activity by monitoring echolocation calls using SM3 bat detectors (Wildlife Acoustics, Inc., Maynard, MA, USA). Three sites ran concurrently. Bat detectors were set to record activity using triggers from 30 min before sunset on the first night until 30 min after sunrise on the ninth morning. At each site, one bat detector was attached to the middle experimental lighting column 1 m below the lantern, using street sign and Tamtorque sign-fixing clamps, with the microphone on the detector pointing slightly downwards and positioned on the same side of the column as the lantern. Bat detectors were randomized across sites. Files were stored as wavefile audio (WAV) files. The settings on the detectors were: high-pass filter 16 kHz; sample frequency 384 kHz; minimum frequency 16 kHz; maximum frequency 120 kHz; maximum recording time 15 s; trigger level 12 dB.

Bat activity for each lighting level was measured as the number of passes over each two-night period. Each 15 s file containing echolocation calls was considered as one bat pass [36]. At sites 19, 20 and 21 the sub-contractor failed to change the lighting level according to the agreed schedule, so bat passes were only counted for one night per treatment, which was selected at random. To compare bat feeding rates at different light levels, we calculated the buzz ratio, i.e. the proportion of passes that contained a feeding buzz [37] at each lighting level.

At seven sites (one from each of the three recording periods), a 12 megapixel 1080 HD Hunting Trail Infra-Red Camera (SpyCameraCCTV, Bristol, BS5 9PQ, UK) was attached to the lighting column to estimate the number of insects attracted to each lighting level. Infrared cameras were used so that the number of insects could be estimated when the street lights were dimmed to low light levels (25%) or switched off (0%). The camera takes high-resolution still images (12 megapixels) meaning that even small flies appeared on the images. The camera was attached to the lighting column immediately below the lantern, so its focus was within the light cone. A burst of three still images were taken once an hour throughout the night (sunset until sunrise). These data were used to compare the attractiveness of the LED lights at different lighting levels to aerial insects.

Nightly temperature and humidity were recorded at each site with a Tinytag TGP-4017 Plus 2 Internal Temperature data logger (Gemini Data Loggers UK Ltd., Chichester, PO19 8UJ, UK). Mean nightly rainfall (mm) and wind speed (km hr^−1^) were obtained from Met Office weather stations within 35 km of each site (www.metoffice.gov.uk/).

### 2.2. Data processing

All bat calls were analysed using Kaleidoscope Pro (v. 3.1.1, Wildlife Acoustics, Inc.) with British Bat Classifiers (v. 3.0.0). The auto-identification of *P. pipistrellus* and *P. pygmaeus* was accepted [36]. However, all other calls were manually identified to either species (*Eptesicus serotinus*, *Nyctalus noctula*, *Pipistrellus nathusii* and *Plecotus auritus*) or group (*Myotis* spp.); *Myotis* spp. are usually grouped because of the difficulty of separating the echolocation calls of the different species [38–40]. We also manually identified files that had a margin factor of zero (either Kaleidoscope Pro was unable to identify the call or classified the call as a noise file). Margin scores in Kaleidoscope Pro are uncalibrated confidence scores, whereby higher values are more likely to be correctly identified than lower values. Species identification was verified for 0.5% of the bat echolocation call files (676 files) to ensure that the auto-identification software was working effectively. These files were randomly selected across all sites to account for any differences between sites and included noise files to ensure that all files that contained a bat pass were being included in the analysis.

As we did not manually verify species from every file, we calculated the feeding buzz from a representative sample of files. For each site, we separated calls for each lighting level, then randomly selected 5% of files to check if a feeding buzz was present (mean number of files per lighting level were 35, 44, 51 and 48 for 0%, 25%, 50% and 100% lighting levels, respectively). We identified all feeding buzzes from all species, but they were mostly from *P. pipistrellus.* All noise files were excluded as a bat pass had to occur for a feeding buzz to be present. We calculated the buzz ratio to determine how the proportion of feeding buzzes compared with the number of echolocation calls changed with light intensity.

Insect activity was determined for one night of each lighting level, when there was no rain; this was owing to the difficulty in identifying the presence of an insect from an image when raining. Each visible white dot on the image was counted as an insect [12]. Only insects that were within the light cone, i.e. directly underneath the light were counted and we excluded non-volant invertebrates, i.e. we did not include spiders, many of which make their webs on street lights [41]. It was only possible to estimate total insect abundance and not to identify species. The number of insects counted in each image was carried out blind, i.e. the scorer was unaware of the lighting level when counting the number of insects. The number of insects from the three images for each hour was averaged and the hourly totals then averaged over the night for each lighting level. This reduced ‘noise’ that might be introduced if any of the three images were unclear.

### 2.3. Statistical analyses

Data were analysed in R Studio using R v. 3.3.3 (R Core Team 2017). We used generalized linear mixed models (GLMMs) to determine potential drivers of bat activity, insect counts and buzz ratios using the lme4 package [42]. Models for bat activity and insect counts followed a negative binomial distribution with a log-link function, and the model for buzz ratio followed a binomial distribution with a logit-link function. Model choice was based on backward selection based on the second-order information criterion (AICc) using the bbmle package [43]. If the *Δ*AICc was less than 2 between models, we chose the model with the fewest number of parameters [44]. Model fit was validated using the Dharma package [45] to ensure that data were not overdispersed and to provide plots of residuals. Before fitting the GLMMs, we checked to see that the predictors, particularly the weather variables, were not correlated i.e. Spearman's rank correlation coefficient less than 0.5 [46].

For bat activity (bat passes), we used three models; all species, *P. pipistrellus* and *Myotis* spp. For all three models, the fixed factors included lighting level (0%, 25%, 50% and 100%) as well as standardized weather variables (centred around a mean of 0 and a standard deviation of 1), mean nightly temperature (°C), mean nightly wind speed (km hr^−1^) and mean nightly rainfall (mm). Site was included as a random effect to account for repeated measurements within each lighting column. Date was also included as a random effect to account for recording at multiple sites (three sites concurrently). Post hoc comparisons between intermediate lighting levels (i.e. 25% versus 50%, 25% versus 100% and 50% versus 100%) were carried out using the multcomp package [47] with single-step corrected probabilities.

The coefficient of determination (*R*^2^) was calculated to compare the goodness-of-fit across the models for different bat species [48]. In mixed-effect models, *R*^2^ has two classifications: marginal, which is the proportion of variance in the response variable explained by the fixed effects, and conditional, which is the proportion of variance in the response variable explained by both the fixed and random effects [49]. *R*^2^ values for the buzz ratio model were calculated using the MuMIn package [50], and the *R*^2^ values for the bat activity and insect count models were calculated as proposed by Nakagawa *et al*. [51].

## 3. Results

Across 21 sites, we recorded 135 228 files that included 74 965 bat passes from seven species/species groups. Most passes (76.7%) were from *P. pipistrellus*, followed by *P. pygmaeus* (20.9%), *N. noctula* (1.9%), *Myotis* spp. (0.2%), *Eptesicus serotinus* (0.08%), *Plecotus auritus* (0.08%) and *P. nathusii* (0.08%) (electronic supplementary material, tables S1–S4). No other species were recorded. From the 676 files that were manually verified, there was 87% agreement between the manual and automatic classifications, with 100% agreement with the automatic classifications of *P. pipistrellus* and *P. pygmaeus*. Kaleidoscope occasionally classified a file as a noise file or was unable to determine a classification, even when a call was present. As all files that were not classified as *P. pipistrellus* or *P. pygmaeus* were manually identified, we feel that our method was appropriate, given the large amount of data collected and the time needed to analyse all the data manually.

Across the 21 sites, mean light intensities for each lighting level were 11.35 lux (s.d. 3.23, range 8.68–14.9 lux) for 25%, 20.23 lux (s.d. 3.23, range 16.77–23.9 lux) for 50% and 35.46 lux (s.d. 5.94, range 29.4–44.0 lux) for 100%.

Statistical analyses were carried out on the number of bat passes for all species, *P. pipistrellus*, *Myotis* spp., feeding behaviour (buzz ratio) and mean insect counts, with standardized weather variables included as fixed factors in the GLMMs. The best models, determined by the lowest AICc values, generally included temperature (°C) and wind speed (km hr^−1^) but not mean nightly rainfall (mm). Temperature had a positive significant effect on the number of bat passes, i.e. there were more bat passes as the nightly temperature increased, whereas wind speed had a significant negative effect on the number of bat passes, i.e. there were fewer bat passes as the nightly wind speed increased. So, it was important that both variables were included as fixed effects in the model.

**Figure 2.**
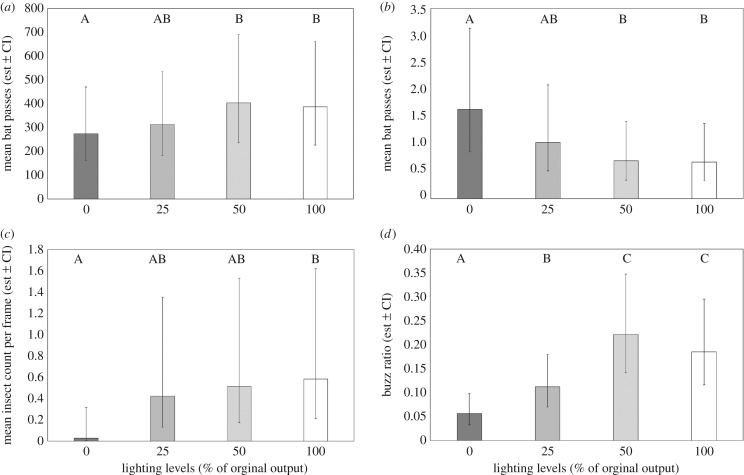
Mean predicted bat activity (number of bat passes) back-transformed across all sites (*n *= 21) for each lighting level for (*a*) *Pipistrellus pipistrellus* and (*b*) *Myotis* spp. (*c*) Mean predicted insect counts back-transformed across selected sites (*n *= 7) for each lighting level. (*d*) Mean predicted buzz ratios back-transformed across all sites (*n *= 21) for each lighting level. For all graphs letters identify groups that were significantly different from each other and vertical lines denote 95% confidence intervals (CIs).

When considering all bat species, there were significantly more bat passes at 50% compared to 0% lighting levels, but not between 25% or 100% and 0% levels (table 1). For light-opportunistic *P. pipistrellus,* the results were broadly similar: there were significantly more passes at 50% and 100%, compared with the 0% lighting level, but there was no difference in the number of bat passes between the 0% and 25% lighting levels (table 1 and figure 2*a*). Conversely, higher light intensities had a negative effect on the light-averse *Myotis* spp. There were significantly fewer *Myotis* passes at 50% and 100% lighting levels, compared with the unlit treatment, but there was no significant difference between the 0% and 25% lighting levels (table 1 and figure 2*b*).

**Table 1.** Results from GLMMs for the bat passes of (*a*) all species, (*b*) *Pipistrellus pipistrellus* and (*c*) *Myotis* spp., (*d*) buzz ratios for all species (based on a 5% sample) and (*e*) mean insect counts. (All estimates were compared against the unlit treatment (0%). Significant results are in bold. *p < 0.05, **p < 0.01, ***p < 0.001.)

model	estimate	s.e.	*Z*-value	*p*-value	marginal *R*^2^	conditional *R*^2^
(*a*) all species					0.183	0.832
25%	0.174	0.164	1.059	0.289		
**50%**	**0**.**391**	**0**.**161**	**2**.**433**	**0**.015*		
100%	0.290	0.160	1.810	0.070		
**temperature (^o^C)**	**0**.**473**	**0**.**093**	**5**.**084**	<**0**.001***		
**wind speed (km hr^−1^)**	−**0**.**191**	**0**.**074**	−**2**.**572**	**0**.010*		
(*b*) *P. pipistrellus*					0.203	0.851
25%	0.130	0.169	0.767	0.443		
**50%**	**0**.**386**	**0**.**168**	**2**.**304**	**0**.021*		
**100%**	**0**.**343**	**0**.**167**	**2**.**054**	**0**.040*		
**temperature (^o^C)**	**0**.**531**	**0**.**097**	**5**.**452**	<**0**.001***		
**wind speed (km hr^−1^)**	−**0**.**252**	**0**.**079**	−**3**.**207**	**0**.001**		
(*c*) *Myotis* spp.					0.126	0.797
25%	−0.408	0.231	−1.771	0.077		
**50%**	−**0**.**828**	**0**.**237**	−**3**.**501**	<**0**.001***		
**100%**	−**0**.**740**	**0**.**242**	−**3**.**057**	**0**.002***		
rain (mm)	−0.340	0.184	−1.844	0.065		
wind speed (km hr^−1^)	−0.201	0.111	−1.861	0.063		
(*d*) buzz ratio					0.061	0.196
**25%**	**0**.**689**	**0**.**217**	**3**.**170**	**0**.001**		
**50%**	**1**.**371**	**0**.**218**	**6**.**292**	<**0**.001***		
**100%**	**1**.**190**	**0**.**220**	**5**.**406**	<**0**.001***		
**temperature (^o^C)**	**0**.**427**	**0**.**168**	**2**.**540**	**0**.011*		
(*e*) insect counts					0.188	0.227
25%	2.686	1.422	1.888	0.059		
50%	2.729	1.423	1.917	0.055		
**100%**	**2**.**905**	**1**.**415**	**2**.**053**	**0**.040*		

The insect count data also showed significantly higher insect activity at the 100% lighting level compared with the unlit treatment, but there was no difference between 0% and 25% or 50% lighting levels (table 1 and figure 2*c*). There were significantly more feeding buzzes at 25%, 50% and 100% lighting levels, compared with the unlit treatment (table 1 and figure 2*d*).

While there were no significant differences between intermediate light levels, i.e. 25% compared to 50% or 100%, or 50% compared to 100% (table 2) for the bat activity data for any of the species or insect counts, there were significantly more feeding buzzes at 50% and 100%, compared with the 25% lighting level (table 2).

**Table 2.** Results of the post-hoc comparisons applied to GLMMs for the bat passes of (*a*) all species, (*b*) *Pipistrellus pipistrellus* and (*c*) *Myotis* spp., (*d*) buzz ratios for all species (based on a 5% sample) and (*e*) mean insect counts. (Lighting levels were 25 (25%), 50 (50%) and 100 (100%). Significant results are in bold. *p < 0.05, **p < 0.01, ***p < 0.001.)

model	estimate	s.e.	*Z*-value	*p*-value
(*a*) all species				
50–25	0.217	0.161	1.343	0.536
100–25	0.116	0.159	0.727	0.886
100–50	−0.101	0.157	−0.641	0.919
(*b*) *P. pipistrellus*				
50–25	0.257	0.167	1.535	0.416
100–25	0.213	0.164	1.298	0.564
100–50	−0.043	0.163	−0.265	0.994
(*c*) *Myotis* spp*.*				
50–25	−0.420	0.257	−1.635	0.358
100–25	−0.332	0.265	−1.253	0.592
100–50	0.088	0.271	0.325	0.988
(*d*) buzz ratio				
**50–25**	**0**.**682**	**0**.**163**	**4**.**192**	<**0**.001***
**100–25**	**0**.**501**	**0**.**161**	**3**.**116**	**0**.010**
100–50	−0.181	0.161	−1.125	0.670
(*e*) insect counts				
50–25	0.043	0.723	0.059	1.000
100–25	0.219	0.696	0.315	0.988
100–50	0.177	0.689	0.257	0.994

## 4. Discussion

Our results are broadly consistent with our hypotheses, that higher light levels (50% and 100%) increased the activity of light-opportunistic species such as *P. pipistrellus*, but reduced the activity of light-averse species such as *Myotis* spp. However, lower light levels (25%) do not affect activity levels of either light-opportunistic or light-averse species of bats compared to the unlit treatment (0%).

The increase in the number of bat passes of the light-opportunistic *P. pipistrellus* at 50% and 100%, compared to the unlit treatment, is most probably owing to the greater number of insects being attracted to the street lights at higher lighting levels. This supports the attraction-by-insects hypothesis, as opposed to the attraction-by-artificial-light hypothesis, which argues that bats are attracted to the lights for other reasons [14]. Foraging benefits can also be inferred from the buzz ratio data. The proportion of feeding buzzes compared to the number of bat passes was significantly higher at the 25%, 50% and 100% lighting levels than the unlit treatment. Also, there were significantly more buzzes relative to echolocation calls at the 50% and 100% lighting levels compared to the 25% level. Our feeding buzz data suggest that the main benefit for some species of bats flying close to street lights is to prey on the insects attracted to the light source. Even though the number of light-opportunistic bat passes did not increase significantly at the 25% lighting level, compared to the unlit treatment, nor between intermediate lighting levels (i.e. 25% and 50% or 25% and 100%), the buzz ratios increased, suggesting that these species of bats increase their feeding efficiency at street lights. This could be owing to the reduced anti-predator behaviour of moths [52] or because around street lights bats may possibly feed on large numbers of relatively small insects that have a lower energy content than larger insects.

Furthermore, there were significantly more insects at the 100% compared to the unlit treatment and the differences between the 25% and 50% lighting levels and the unlit treatment were almost significant (table 2). While there were not significantly more insects at the 25% or 50% lighting levels compared to the unlit treatment, there were more feeding buzzes relative to the number of bat passes. This could be owing to the absence of a linear relationship between the number of insects attracted to a light source and its illuminance [53]. Although the light intensity at the 50% level (mean 20.23 lux) was double that of the 25% level (mean 11.35 lux), this does not mean that double the number of insects should be attracted to the 50% lighting level. To determine the attractiveness of a light source, it is necessary to consider the spectral sensitivities of the insects [3] and calculate either the square root of the ratio between the illuminance of the light source and its surrounding background [54] or use a function of the luminance of the light source [55]. The difference between the insect and buzz ratio data could also be owing to the smaller sample sizes for the insect counts.

Lighting level appeared to have a stronger effect at 50% than 100% for both bat activity and feeding behaviour, possibly because when the LED street lights are at 50% of their original output, there is an increase in insect numbers and hence feeding opportunities but fewer risks from potential predators. Alternatively, when light intensities increase above 50% of the original output, the illuminance may disturb bats [56] or, at light intensities above 50%, more bats may be attracted to the higher insect numbers, and hence be affected by echolocation interference from the calls of other bats. This makes it more difficult for a bat to differentiate its own returning echoes from those of conspecifics [57].

It is unsurprising that we found significantly fewer bat passes of *Myotis* spp. at 50% and 100% lighting levels compared to the unlit treatment [26,33]. However, it is encouraging that the low lighting level (25%) did not have a detrimental effect on the number of *Myotis* spp. passes. From a conservation perspective, this is a positive outcome as it means there is scope to work with local authorities to determine if it is possible to find a light intensity that is acceptable for humans but does not adversely affect bat activity, particularly for light-averse species.

At the low lighting level (25%), as less light was distributed from the light source, it is likely that dark corridors were created that light-averse species, such as *Myotis* spp., could fly along, either as a more efficient commuting route or even to forage. However, once the street light intensities exceeded 11.35 lux, the perceived threat of predation becomes too great, significantly reducing the number of *Myotis* spp. passes near the street lights. This contrasts with an earlier study, which found that LED light intensities as low as 3.6 lux negatively affected the number of bat passes from light-averse bats such as *Myotis* spp. and *Rhinolophus hipposideros* [26]. This could be owing to differences in experimental design: our study took place in suburban areas, where street lights have existed for decades, and hence the bats may have adapted to the presence of artificial lights, whereas the earlier study set up street lights in unlit areas [26], and hence the novelty of lighting may have affected the bats differently. Differences could also be because fewer *Myotis* spp. are found in suburban areas compared to rural areas (figure 2*a*,*b*). As *Myotis* spp. are light-averse, they tend to avoid suburban areas when commuting and foraging, preferring more cluttered habitats [17,19]. Our results are consistent with an earlier study which also found that light intensity had a significant positive effect on light-opportunistic species such as *P. pipistrellus*, but a significant negative effect on light-averse species such as *Myotis* spp. [33].

Reducing the light intensities of street lights could also benefit invertebrates by decreasing flight-to-light behaviour, thereby lowering the risk of mortality from exhaustion and predation, as well as preventing disruptions to biological cycles [58,59]. To reduce the ecological impact on invertebrates, it has been advised that LED street lights should be dimmed to 50% of their original output (less than 14 lux) and adhere to a part-night lighting scheme, i.e. switched off between midnight and 04.00 [60].

In conclusion, our results support dimming as an effective strategy to mitigate the ecological impacts of street lights as it seems possible to achieve a light intensity that could benefit both light-opportunistic and light-averse species of bats [56], potentially realigning the balance that existed before street lighting dominated our landscapes. It is worth mentioning that ideally the installation of street lights should be avoided, but as this is not feasible in many areas owing to safety and security reasons, dimming seems to be the most suitable alternative.

We believe further studies are required to investigate the impacts of dimming in different locations to include other light-averse species, such as *Plecotus* and *Rhinolophus* species. It would also be useful to repeat this study, using residential areas, instead of A roads where street lights are typically 5 m as opposed to 10 m high, and have a lower power and illuminance. It might be possible to reduce light intensities even further, while still striking the balance between maintaining biodiversity, economic benefits and human safety [61].

## Supplementary Material

Table S1

## Supplementary Material

Table S2

## Supplementary Material

Table S3

## Supplementary Material

Table S4

## Data accessibility

The datasets supporting this article have been uploaded as part of the electronic supplementary material.
