# Supplementary material for: Effects of dimming light-emitting diode street lights on light-opportunistic and light-averse bats in suburban habitats
Source: R Soc Open Sci. 2018 Jun 6;5(6):180205. doi: 10.1098/rsos.180205 (PMC6030271; doi:10.1098/rsos.180205)
Supplement: Table S1 [file rsos180205supp1.docx]

Table S1. The number *Pipistrellus pipistrellus* bat passes at each site over the two recording nights for the four lighting levels (0%, 25%, 50% and 100%), except for sites 19, 20 and 21 (marked with an asterisk) where only one night’s data were used.

| Site | No. of passes: 0% | No. of passes:  25% | No. of passes: 50% | No. of passes: 100% |
| --- | --- | --- | --- | --- |
| 1 | 38 | 2 | 34 | 51 |
| 2 | 323 | 10 | 46 | 52 |
| 3 | 1674 | 1274 | 1142 | 1173 |
| 4 | 293 | 573 | 385 | 370 |
| 5 | 24 | 34 | 138 | 31 |
| 6 | 531 | 225 | 50 | 281 |
| 7 | 291 | 135 | 128 | 84 |
| 8 | 1198 | 1533 | 2212 | 1646 |
| 9 | 82 | 117 | 146 | 78 |
| 10 | 58 | 96 | 63 | 220 |
| 11 | 1504 | 2045 | 1975 | 2123 |
| 12 | 167 | 491 | 1319 | 378 |
| 13 | 116 | 115 | 1072 | 742 |
| 14 | 201 | 60 | 267 | 171 |
| 15 | 1018 | 1695 | 2903 | 1545 |
| 16 | 940 | 1211 | 2024 | 1317 |
| 17 | 398 | 1385 | 598 | 1483 |
| 18 | 1673 | 2065 | 2031 | 2818 |
| 19* | 383 | 266 | 355 | 400 |
| 20* | 93 | 464 | 143 | 261 |
| 21* | 88 | 164 | 97 | 112 |
