# Supplementary material for: Effects of dimming light-emitting diode street lights on light-opportunistic and light-averse bats in suburban habitats
Source: R Soc Open Sci. 2018 Jun 6;5(6):180205. doi: 10.1098/rsos.180205 (PMC6030271; doi:10.1098/rsos.180205)
Supplement: Table S2 [file rsos180205supp2.docx]

Table S2. The number *Myotis* spp. bat passes at each site over the two recording nights for the four lighting levels (0%, 25%, 50% and 100%), except for sites 19, 20 and 21 (marked with an asterisk) where only one night’s data were used.

| Site | No. of passes: 0% | No. of passes:  25% | No. of passes: 50% | No. of passes: 100% |
| --- | --- | --- | --- | --- |
| 1 | 5 | 0 | 1 | 2 |
| 2 | 1 | 0 | 0 | 0 |
| 3 | 0 | 0 | 0 | 0 |
| 4 | 2 | 1 | 1 | 1 |
| 5 | 0 | 0 | 0 | 1 |
| 6 | 5 | 3 | 2 | 1 |
| 7 | 2 | 2 | 2 | 0 |
| 8 | 4 | 2 | 1 | 2 |
| 9 | 0 | 0 | 0 | 0 |
| 10 | 9 | 7 | 8 | 1 |
| 11 | 0 | 0 | 0 | 0 |
| 12 | 0 | 0 | 0 | 0 |
| 13 | 3 | 3 | 3 | 4 |
| 14 | 2 | 1 | 0 | 1 |
| 15 | 1 | 1 | 0 | 0 |
| 16 | 7 | 7 | 5 | 7 |
| 17 | 4 | 0 | 0 | 2 |
| 18 | 5 | 1 | 1 | 0 |
| 19* | 11 | 20 | 8 | 8 |
| 20* | 2 | 0 | 1 | 1 |
| 21* | 5 | 0 | 0 | 1 |
