# Supplementary material for: Effects of dimming light-emitting diode street lights on light-opportunistic and light-averse bats in suburban habitats
Source: R Soc Open Sci. 2018 Jun 6;5(6):180205. doi: 10.1098/rsos.180205 (PMC6030271; doi:10.1098/rsos.180205)
Supplement: Table S3 [file rsos180205supp3.docx]

Table S3. Mean insect counts (SD) at each site for the four lighting levels (0%, 25%, 50% and 100%) for one recording night.

| Site | Mean insect count: 0% | Mean insect count: 25% | Mean insect count: 50% | Mean insect count: 100% |
| --- | --- | --- | --- | --- |
| 1 | 0 (0) | 0.07 (0.22) | 0.26 (0.36) | 0.52 (0.44) |
| 6 | 0.03 (0.12) | 0.03 (0.12) | 0.12 (0.25) | 0.76 (1.17) |
| 9 | 0.22 (0.46) | 0.41 (0.62) | 0.19 (0.47) | 0.52 (1.08) |
| 11 | 0 (0) | 0.89 (1.20) | 0.22 (0.46) | 1.04 (1.40) |
| 14 | 0 (0) | 0.04 (0.12) | 0 (0) | 0.67 (1.05) |
| 18 | 0 (0) | 1.85 (2.69) | 2.52 (3.07) | 2.00 (2.21) |
| 21 | 0 (0) | 0.45 (0.65) | 0.82 (0.92) | 0.03 (0.12) |
