# Supplementary material for: Effects of dimming light-emitting diode street lights on light-opportunistic and light-averse bats in suburban habitats
Source: R Soc Open Sci. 2018 Jun 6;5(6):180205. doi: 10.1098/rsos.180205 (PMC6030271; doi:10.1098/rsos.180205)
Supplement: Table S4 [file rsos180205supp4.docx]

Table S4. The buzz ratio for all bat species at each site over the two recording nights for the four lighting levels (0%, 25%, 50% and 100%), except for sites 19, 20 and 21 (marked with an asterisk) where only one night’s data were used.

| Site | Buzz ratio: 0% | Buzz ratio: 25% | Buzz ratio: 50% | Buzz ratio: 100% |
| --- | --- | --- | --- | --- |
| 1 | 1.00 | 0.00 | 0.5 | 0.00 |
| 2 | 0.11 | 0.00 | 0.00 | 0.00 |
| 3 | 0.58 | 0.19 | 0.25 | 0.19 |
| 4 | 0.05 | 0.11 | 0.25 | 0.22 |
| 5 | 0.00 | 0.00 | 0.10 | 0.00 |
| 6 | 0.00 | 0.06 | 0.00 | 0.04 |
| 7 | 0.06 | 0.00 | 0.00 | 0.00 |
| 8 | 0.10 | 0.12 | 0.14 | 0.16 |
| 9 | 0.00 | 0.20 | 0.25 | 0.00 |
| 10 | 0.00 | 0.22 | 0.00 | 0.41 |
| 11 | 0.05 | 0.36 | 0.41 | 0.44 |
| 12 | 0.08 | 0.31 | 0.37 | 0.40 |
| 13 | 0.00 | 0.00 | 0.42 | 0.26 |
| 14 | 0.10 | 0.00 | 0.11 | 0.05 |
| 15 | 0.11 | 0.13 | 0.21 | 0.05 |
| 16 | 0.02 | 0.11 | 0.26 | 0.15 |
| 17 | 0.13 | 0.31 | 0.26 | 0.40 |
| 18 | 0.04 | 0.18 | 0.27 | 0.23 |
| 19* | 0.20 | 0.00 | 0.07 | 0.11 |
| 20* | 0.08 | 0.18 | 0.00 | 0.20 |
| 21* | 0.00 | 0.10 | 0.00 | 0.00 |
